# Research on rheological behavior of fresh concrete single-cylinder pumping based on SPH-DEM

**DOI:** 10.1038/s41598-023-45702-2

**Published:** 2023-11-27

**Authors:** Wanrong Wu, Wang Chen, Guoyi Lu, Jiaqian Wang, Guangtian Tian, Boxuan Xu, Chao Deng

**Affiliations:** 1 College of Mechanical and Electrical Engineering, Central South University, Changsha, 410083 China; 2 State Key Laboratory of Key Technologies of Lifting Machinery, ZoomLion, Changsha, 410803 China

**Keywords:** Engineering, Civil engineering

## Abstract

In contrast to traditional approaches to simulating fresh concrete, the model applied here allows issues such as liquid phase and the motion of sub-scale particles to be considered. The rheological behavior of fresh concrete materials was investigated, and the slump test and pumping process of fresh concrete were simulated by combining the smooth particle hydrodynamics coupled with discrete element method. Based on Bi-viscosity model and Bingham model, linear and nonlinear fitting of rheometer data and the derivation equations were educing. Bi-viscosity model and the Bingham model were compared in slump test. The results show that the Bi-viscosity model is more accurate in simulation, and the error percentage is less than 10%. The Bi-viscosity model was used to simulate and predict the results of slump experiment, and the influence of rheological parameters on the slump velocity and shape was obtained. The simulation analysis model of concrete single-cylinder pumping is established, and the experimental and simulation analysis models are compared. The results show that the SPH-DEM pumping pressure prediction is very close to the experimental results.

## Introduction

Despite the development of computational science and simulation technology, some physical phenomena, can be reasonably characterized and analysed by computer technology. However, for the transport^1–3^, separation^4,5^, water secretion, and blockage^6–8^ of the multiphase components of fresh concrete (concrete for short), results can only be obtained experimentally, and it is difficult to perform qualitative analysis, evaluation, and prediction. Due to the complex composition of concrete, the experimental process is difficult to analyse and control more accurately the specific influencing factors^9^ for the changes in pumpable performance.

Roussel et al.^10,11^ argued that fresh concrete can be used to show the properties of concrete with Bingham fluid, which is a non-Newtonian fluid with a certain yield stress, and irreversible flow and deformation will occur only when the fluid is subjected to a shear stress greater than the yield stress. As the research progresses, and the fitness ratio of concrete changes, the shear rate of fresh concrete is not linearly related to the shear stress similar to the Bingham model, but will exhibit shear thinning and shear thickening properties^12^. Checking the pumpability of concrete, in engineering applications, is generally predicted by slump tests. Chidiac et al.^13^ showed in their study that the slumping process of concrete occurs only when the gravity of the bottom material exceeds the effect of yield stress, while no shear flow occurs in the upper region, and the bottom concrete keeps flowing downward, resulting in a continuous increase in experimental slump and eventually The flow stops when the shear stress from the gravity of the top concrete is equal to the yield. However, the experimental procedure for concrete cannot quantify its characteristics such as viscosity, velocity, and yield, and it is difficult to analyze the factors affecting the concrete pumping process.

Bingham model is commonly used to express the rheological properties of concrete^14^, which is $\tau = \tau_{y} + \mu_{p} \dot{\gamma }$ with two rheological parameters, yield stress $\tau_{y}$ and plastic viscosity $\mu_{p}$. Due to the change of concrete proportioning and properties, the linear relationship of Bingham model at this stage is difficult to characterize all concrete stress–strain relationships. Subsequent studies have improved the linear relationship of the Bingham model, making the model retain the original yield stress with the addition of a nonlinear fit^15^, i.e., $\tau = \tau_{y} + K\left( {\dot{\gamma }} \right)\dot{\gamma }^{n}$, which adds a power-law index $n$ that allows the model to characterize shear thinning or shear thickening phenomena. The classical computational fluid dynamics (CFD) approach treats concrete as a single fluid phase, ignoring the coarse aggregates, reinforcing fibers and other large scale particles in it, and calculates the concrete mesh flux to obtain the velocity variation and shear deformation region of the concrete flow process^16^. Robin De Schryver et al.^17^ used OpenFOAM (OpenFOAM Fundation Ltd, incorporated in England) single-phase flow to calculate the pressure of the concrete pumping process, the results are one pressure value higher than the experimental values, which is explained by the fact that concrete undergoes compositional changes during transport in the pumping pipeline, which results in much smaller rheological parameters near the walls than the actual concrete.Secrieru et al.^18^ and others, using the commercial software Fluent (Ansys Ltd), divided the concrete into two phases, using the rheological parameters of the lubrication layer near the wall and the internal plunger flow using the rheological parameters of the concrete itself for pumping pressure analysis, and the simulation results were very close to the experimental numerical results.

The phenomenon of formation and analysis out of the pumping process can also be used Discrete element method (DEM), which discrete the concrete into two phases of aggregate, slurry, and fitted with different material friction and recovery coefficients, respectively. Cui et al.^19^, discrete concrete large scale particles and continuous fluid, analyzed the concrete coarse aggregate shape for concrete test L box clogging factors were explored. Krenzer et al.^20^ divided concrete into wet and dry particles, and applied particle replacement method to form a new material particle after wet and dry collision to simulate the aggregation of slurry and aggregates during concrete mixing, and conducted an analytical comparison between slump experiments and simulation to verify the feasibility of DEM method to study concrete materials. However, no systematic industrial application of the DEM method in and pipeline conveying has been seen.

Another analysis method is to use CFD-DEM (particle size small than grid). For example, Zhan et al.^21^ analyzed the trajectory of the particle motion in the pumping pipe and its exploration of the easy clogging parts of the pipe, and the results showed that the particles at the bends are prone to aggregation, and this result is consistent with the results analyzed by Jiang et al.^22^ Considering the large grid size (large particle size), it is difficult to capture the action and characteristics of the boundary layer, which affects the final pressure calculation results.

In summary, the former research method of DEM is more for concrete materials and cannot characterize the fluid properties of concrete, so it is rarely involved in pumping aspects. The classical CFD method, which esteemed the concrete components as single-phase fluid, is far from the actual complex concrete composition. It is impossible to characterize the movement of large-grained aggregates in concrete. In addition, the interaction between aggregate and slurry cannot be characterized, and the causes of aggregate precipitation, settlement and blockage in the pumping process cannot be analyzed. It is necessary to couple the movement of slurry and aggregate. Therefore, the SPH-DEM coupling analysis method is selected here, which can consider the particle motion and the large rigid body motion of the concrete cylinder.

The smoothed particle hydrodynamics coupled with discrete elements method(SPH-DEM) is a sub-particle scale (SPS) analysis method, which can simulate large-scale particle motion (particle size larger than the grid)^23,24^ compared to the classical computational fluid dynamics (CFD) method. The SPH-DEM method is used to establish a concrete model for the pumping rheological behavior of concrete, and the pumping experiments are used to test the applicability of the method and provide evaluation, prediction and improvement of analysis methods for pumping experimental results.

## Methodology

### SPH method

Fresh concrete is a multi-component complex material. The scale can be divided into two phases, namely, large-scale coarse aggregate solid phase and small-scale fluid phase. Smoothed particle hydrodynamics (SPH)^25–27^ is used to solve the fluid phase, which is a computational fluid dynamics method under the Lagrangian framework. In SPH, ‘particles’ represent the fluid space domain. The solution of SPH is solved by the integral and differential of the flow field variables (such as velocity/pressure, etc.) and the kernel function. The Navier–Stokes (N–S) equations of computational fluid dynamics, which continuity and momentum equations are written as Eqs. (1) and (2)

1 $$\frac{d\rho }{{dt}} = - \rho \nabla \cdot U$$

2 $$\frac{dU}{{dt}} = - \frac{1}{\rho }\nabla p + {\Gamma } + g$$

where $\rho$ is fluid density, $p$ is fluid pressure, $U = \left( {U\_x,U\_y,U\_z} \right)$ is velocity vector, $g = \left( {g\_x,g\_y,g\_z} \right)$ is gravitational acceleration, ${\Gamma } = \mu \nabla^{2} U$ is viscous dissipation term. In SPH method, ${\Gamma }$ can be characterized in two ways, artificial viscosity^28^ and SPS turbulent viscosity^29^.

The solution of the Eqs. (1) and (2) consists of two main parts, which are the approximation of the kernel function and the approximation of the particle position, respectively. More detailed analysis of the kernel function can be found in the literature^30,31^. The integral of the function is transformed into the integral of the kernel function written as

3 $$f\left( x \right) = \mathop \smallint \limits_{\Omega } f\left( {x{\prime} } \right)W\left( {x - x{\prime} ,h} \right)dx{\prime}$$

**Figure 1:**
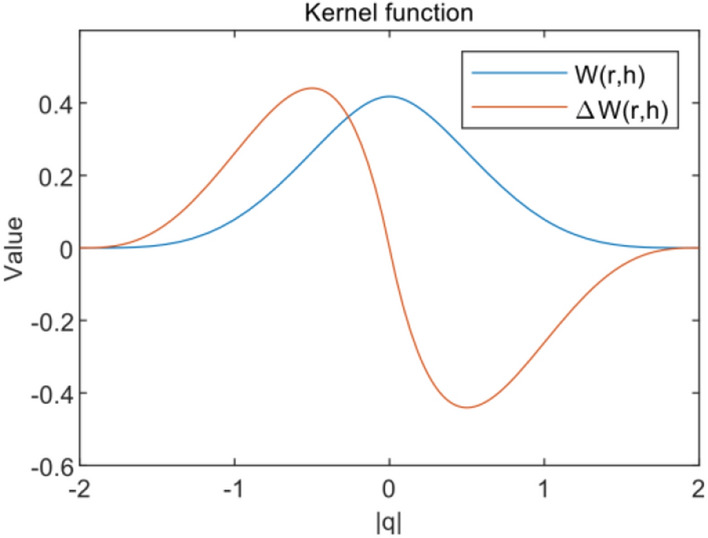
Kernel function.

Where ${\Omega }$ is the computational domain, $W$ is the kernel function, which decreases monotonically with distance, and $h$ is the smooth kernel length, which determines the range of action of the kernel function, which corresponds to the discrete space as

4 $$f\left( {x_{i} } \right) = \mathop \sum \limits_{j = 1}^{N} \frac{{m_{j} }}{{\rho_{j} }}f\left( {x_{j} } \right)W_{ij}$$

in Eq. (4), $m_{j}$ is particle mass and $\rho_{j}$ is density. Similarly, the differentiation of the function can be written as

5 $$\nabla \cdot f\left( {x_{i} } \right) = \mathop \sum \limits_{j = 1}^{N} \frac{{m_{j} }}{{\rho_{j} }}f\left( {x_{j} } \right) \cdot \nabla_{i} W_{ij}$$

where $\nabla_{i}$ is the difference value calculated with respect to particle $i$ position. The SPH kernel function used in this study is the Wendland Quintic fifth-order interpolation kernel function

6 $$W\left( {r,h} \right) = \alpha_{D} \left\{ {\begin{array}{*{20}l} {\left( {2 - {\varvec{q}}} \right)^{4} \left( {1 + 2q} \right), \;\;\;\; 0 \le \left| {\varvec{q}} \right| = r/h \le 2} \hfill \\ {0,\;\;\;\; \left| {\varvec{q}} \right| > 2} \hfill \\ \end{array} } \right.$$

where, $\alpha_{D}$ is a numerical value with respect to dimensionality and is taken as $7/4\pi h^{2}$ for the 2-D case and 21/16 $\pi /h^{3}$ for the 3-D case. the image of the kernel function and its corresponding differential counterpart are shown in Fig. 1.

Therefore, the N-S equation in SPH scheme^26^ is written as

7 $$\frac{{d\rho_{a} }}{dt} = \rho_{a} \mathop \sum \limits_{b} \frac{{m_{b} }}{{\rho_{b} }}U_{ab} \cdot \nabla_{a} W_{ab}$$

8 $$\frac{{dU_{a} }}{dt} = - \mathop \sum \limits_{b} m_{b} \left( {\frac{{p_{a} + p_{b} }}{{\rho_{a} \cdot \rho_{b} }}} \right)\nabla_{a} W_{ab} + g + \mathop \sum \limits_{b} m_{b} \left( {\frac{{4\nu_{0} r_{ab} \cdot \nabla_{a} {\text{W}}_{ab} }}{{\left( {\rho_{a} + \rho_{b} } \right) \cdot \left( {r_{ab}^{2} + \eta^{2} } \right)}}} \right)U_{ab} + \mathop \sum \limits_{b} m_{b} \left( {\frac{{\tau_{ij}^{b} }}{{\rho_{b}^{2} }} + \frac{{\tau_{ij}^{a} }}{{\rho_{a}^{2} }}} \right)\nabla_{a} W_{ab}$$

9 $$p = b\left\{ {\left( {\frac{\rho }{{\rho_{0} }}} \right)^{\gamma } - 1} \right\}$$

where, $p_{a}$ is the pressure at particle *a* and $\nabla_{a}$ denotes the gradient with respect to the coordinates of particle *a*. $\gamma$ = 7, $b = c_{0}^{2} \rho_{0} /\gamma$, $\rho_{0}$ is the reference density and $c_{o} = c\left( {\rho_{o} } \right) = \sqrt {\left( {\partial P/\partial \rho } \right)} |_{{\rho_{o} }}$,which is the speed of sound at the reference density.

Considering that when the fluid is in contact with a solid or a boundary, the integration region of the kernel function is not the whole region that the fluid can contain at this time, the range of action of the kernel function will be truncated by the boundary, which will lead to the loss of accuracy of the kernel function of the SPH^31^. To solve this problem the Dynamic boundary condition(DBC) is chosen for the work of this paper, while DBC is used in the study of Newtonian and non-Newtonian fluids^14,32,33^.

DualSPHysics (open-source CFD code based on SPH method) v5.0.51^34–36^ is used to solve computational fluid dynamics. The Project Chrono engine (open-source DEM code) is coupled for collisions and interactions between solid large particles and bottom area, rigid bodies, and other particles.

### DEM

**Figure 2:**
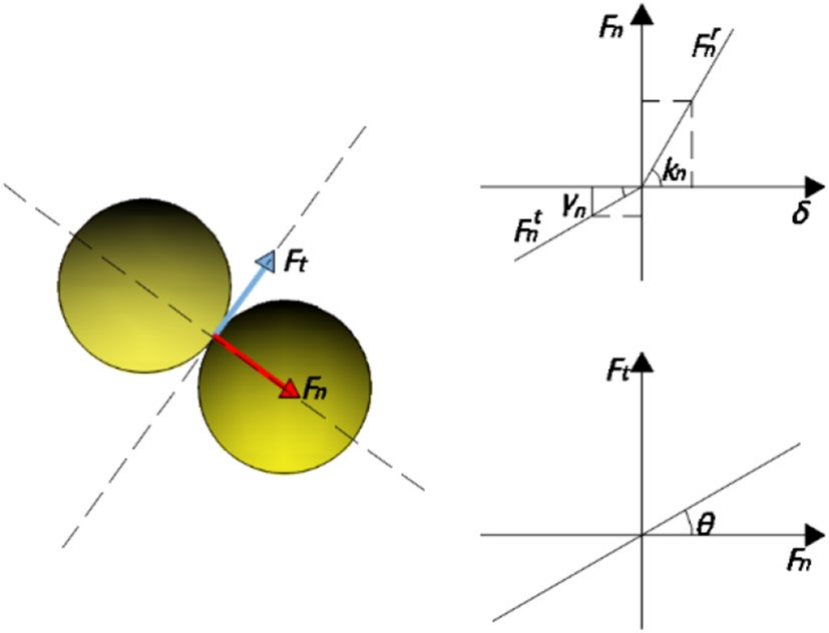
Schematic of DEM.

There are a large number of coarse aggregate particles inside the fresh concrete material. The motion process of large particles is generally solved by DEM method, and then the velocity and displacement are solved by Newton’s law of motion. The solution process of the DEM method is shown in Fig. 2. DEM discretizes the large particles of coarse aggregate in concrete into many small spheres, and obtains the force of the coarse aggregate movement process by solving the collision and deformation between the spheres.

In Fig. 2, the force on the particles can be decomposed into tangential force and normal force. The two forces are composed of the elastic collision force and the damping dissipation force of the material, and the normal force can be obtained from the Coulomb friction law.

10 $$F_{n} = F_{n}^{r} - F_{n}^{t} = k_{n} \delta^{3/2} - \gamma_{n} \delta^{1/2} \dot{\delta }$$

11 $$k_{n} = \frac{4}{3}E^{*} \sqrt {R^{*} } ,\;\;\;\gamma_{n} = - \frac{{lne_{ij} }}{{\sqrt {\pi^{2} + ln^{2} e_{ij} } }}$$

where, $e_{ij}$ is the average elastic collision coefficient of the two materials.

12 $$R^{ *} = \left( {\frac{1}{{R_{1} }} + \frac{1}{{R_{2} }}} \right)^{ - 1} ,\;\;\;E^{ *} = \left( {\frac{{1 - v_{p1}^{2} }}{{E_{1} }} + \frac{{1 - v_{p2}^{2} }}{{E_{2} }}} \right)^{ - 1}$$

where $R_{ij}$ is the particle radius, $E_{ij}$ is the Young 's modulus of the material, and $v_{p1}^{{}}$ is the Poisson 's ratio of the material. For the simplification of the model, the tangential force of large solid particles is taken as a certain proportion of the normal force.

13 $$F_{t} = F_{t}^{r} - F_{t}^{c} = k_{t} \delta^{t} - \gamma_{t} \delta^{t} \dot{\delta }$$

14 $$k_{t} = 2/7k_{n} ,\gamma_{t} = 2/7\gamma_{n} .$$

### Coupled SPH-DEM method

It is also possible to derive the movement of an object by considering its interaction with fluid particles and using these forces to drive its motion. This can be achieved by summing the force contributions for an entire body. By assuming that the body is rigid, the net force on each boundary particle is computed according to the sum of the contributions of all surrounding fluid particles according to the designated kernel function and smoothing length. Each boundary particle k therefore experiences a force per unit mass given by Eq. (15)

15 $$f_{k} = \mathop \sum \limits_{a \in WPs} f_{ka}$$

where,$f_{ka}$ is the force per unit mass exerted by the fluid particle $a$ on the boundary particle $k$, which is given by

16 $$m_{k} f_{ka} = - m_{a} f_{ak}$$

For the motion of the moving body, the basic equations of rigid body dynamics can then be used

17 $$M\frac{dV}{{dt}} = \mathop \sum \limits_{k \in BPs} m_{k} f_{k}$$

18 $$I\frac{{d{{\varvec{\Omega}}}}}{dt} = \mathop \sum \limits_{{k \in B{\varvec{Ps}}}} m_{k} \left( {{\varvec{r}}_{k} - {\varvec{R}}_{0} } \right) \times f_{k}$$

where $M$ is the mass of the object, $I$ the moment of inertia, $V$ the velocity, ${{\varvec{\Omega}}}$ the rotational velocity and ${\varvec{R}}_{0}$ the centre of mass. Equations Eqs. (17) and (18) are integrated in time in order to predict the values of $V$ and ${{\varvec{\Omega}}}$ for the beginning of the next time step. Each boundary particle within the body then has a velocity given by

19 $$u_{k} = V + {{\varvec{\Omega}}} \times \left( {r_{k} - R_{0} } \right).$$

### Rheological model of concrete

Bingham and H-B models were used to study the rheological properties of fresh concrete. Both of them consider the initial flow state of concrete, and the shear stress $\tau$ should be greater than the yield stress $\tau_{y}$ of concrete. The mathematical expression of Bingham model^37^ and H-B model is

20 $$\tau_{B} = \left\{ {\begin{array}{*{20}l} {\tau_{y} + \mu_{p} \dot{\gamma }, \tau \ge \tau_{y} } \hfill \\ {\tau_{y} , \tau < \tau_{y} } \hfill \\ \end{array} } \right.$$

21 $$\tau_{H - B} = \left\{ {\begin{array}{*{20}l} {\tau_{y} + K\left( {\dot{\gamma }} \right)\dot{\gamma }^{n} , \tau \ge \tau_{y} } \hfill \\ {\tau_{y} , \tau < \tau_{y} } \hfill \\ \end{array} } \right.$$

where,$\tau_{B}$ is the shear stress under the Bingham model,$\tau_{y}$ is the yield stress, $\mu_{p}$ is the plastic viscosity, $\dot{\gamma }$ is the shear rate, $\tau_{H - B}$ is the shear stress under the H-B model, $K\left( {\dot{\gamma }} \right)$ is the consistency coefficient, and $n$ is the fitted power law index.

In Eqs. (14) and (15), the part of shear stress less than yield stress is defined by viscosity as

22 $$\mu_{app} = \mathop {\lim }\limits_{{\dot{\gamma } \to 0}} \frac{\tau }{{\dot{\gamma }}} = \infty$$

the viscosity of the motion process is

23 $$\eta = \frac{{\tau_{HBP} }}{{\dot{\gamma }}} = \frac{{\tau_{y} \left( {1 - e^{{ - m\dot{\gamma }}} } \right)}}{{\dot{\gamma }}} + \mu \dot{\gamma }^{n - 1}$$

When the shear rate is small, the viscosity tends to infinity at this time, which will bring convergence problems to the simulation process. Therefore, it is necessary to improve the Eq. (15) reasonably to make the model converge. The improved method used in this paper is to refer to the idea proposed by Papanastious et al.^38^, and make a reasonable correction to convert the H-B model from a discontinuous process to a continuous HBP process, such as

24 $$\tau_{HBP} = \tau_{y} \left( {1 - e^{{ - m\dot{\gamma }}} } \right) + \mu \dot{\gamma }^{n}$$

The viscosity of the motion process at this time is

25 $$\eta = \frac{{\tau_{HBP} }}{{\dot{\gamma }}} = \frac{{\tau_{y} \left( {1 - e^{{ - m\dot{\gamma }}} } \right)}}{{\dot{\gamma }}} + \mu \dot{\gamma }^{n - 1}$$

where,

26 $$\mathop {\lim }\limits_{{\dot{\gamma } \to 0}} \frac{{\left( {1 - e^{{ - m\dot{\gamma }}} } \right)}}{{\dot{\gamma }}} = m$$

**Figure 3:**
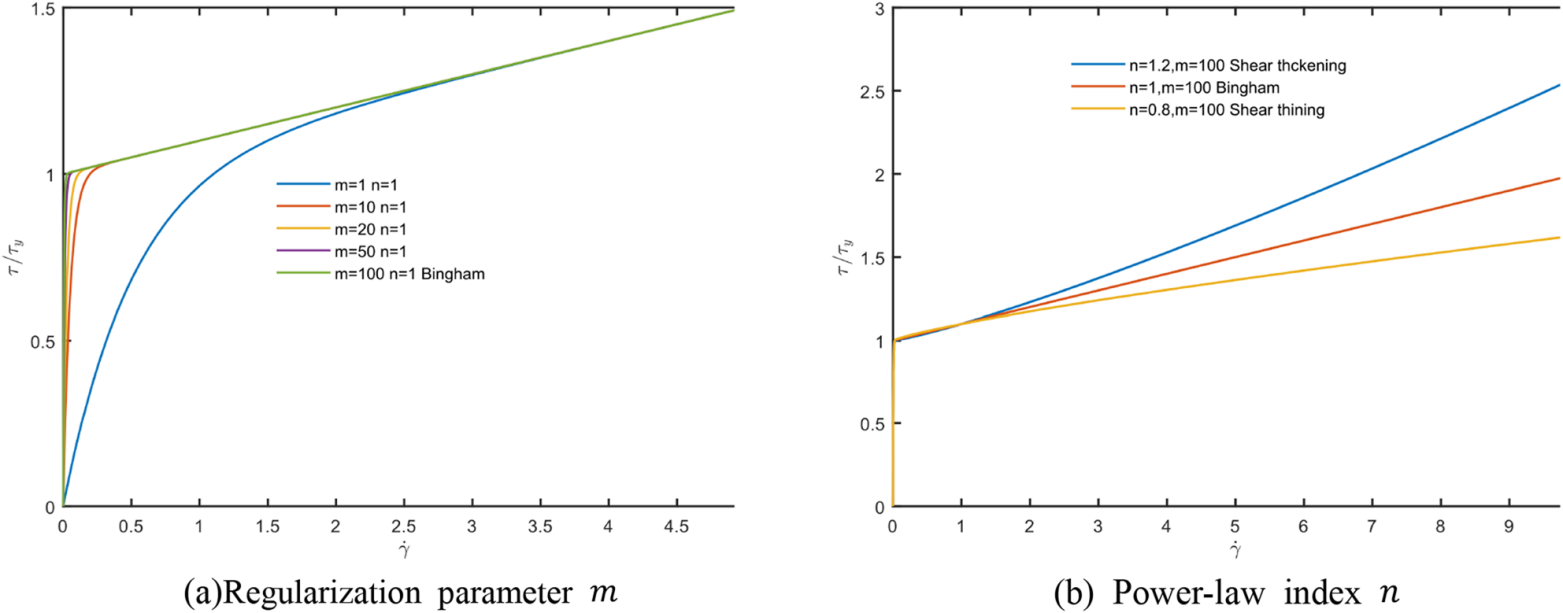
HBP model.

Therefore, the rheological model under the HBP model shows continuity, and as the management parameter m increases, it will become more and more close to the ideal Bingham model, and its mapping relationship and image are shown in Fig. 3.

**Figure 4:**
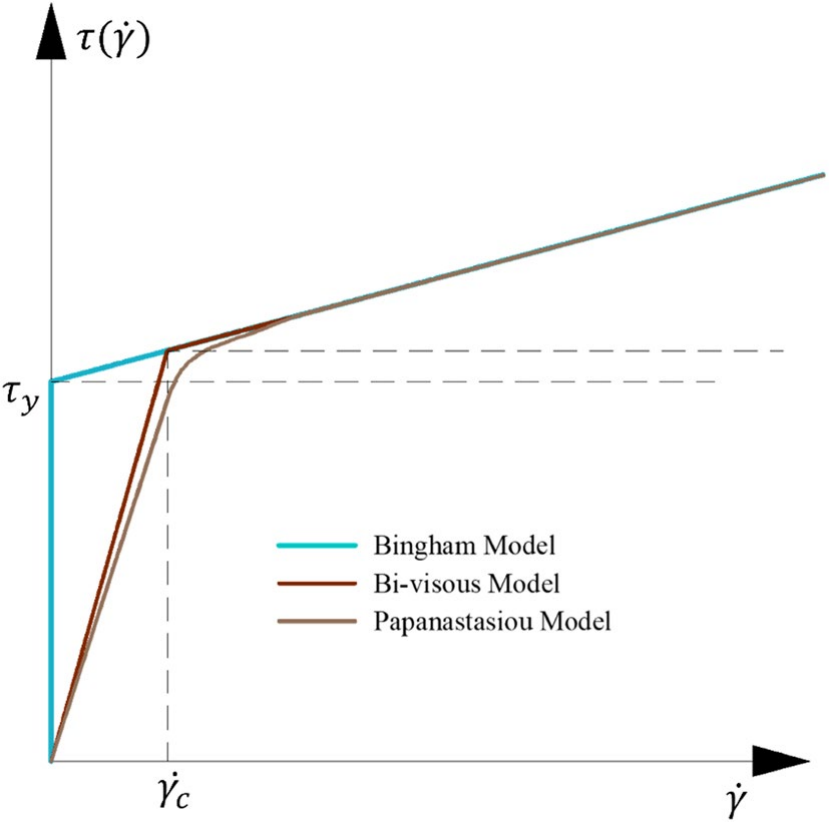
Model comparison.

The Bi-viscosity model is based on the HBP model, which enlarges the viscosity coefficient in the small shear rate region, making the Bi-viscosity model closer to the ideal Bingham model. The image is shown in Fig. 4. It can be seen in the figure that the Bi-viscosity model has a higher degree of fit than the HBP model in the case of small shear rate. In the subsequent research, in order to facilitate the distinction, the Bingham model fitted by the HBP model is called the Bingham model.

### Stress analysis of concrete rheometer

**Figure 5:**
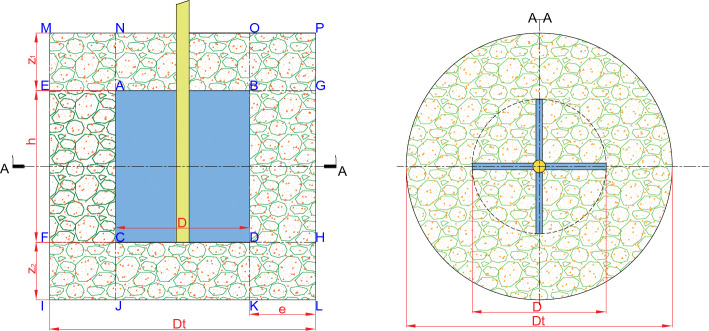
Schematic of rheometer.

The force diagram of the concrete rheometer during the sampling process is shown in Fig. 5. The blade size of the rheometer is fixed, the height of the cylinder is fixed, and the height of the support frame is fixed. It is assumed that the geometric size is AB = $D$, IL = $D_{t}$, FI = $z_{2}$, EF = $h$, ME = $z_{1}$. The areas driven by the rheometer are divided into six areas: ABCD, EFAC-BGHD, ABON, CDKJ, FCJI-DHLK, and MNAE-OPBG. The torques of the above six regions are calculated respectively.

The torque of the rotary process of the rheometer is

27 $$T = T_{1} + T_{2} + T_{3} + T_{4} + T_{5} + T_{6} = \int {rdF}$$

where, $T_{1}$ represents the reaction torque of concrete to the blade in the ABCD region, $T_{2}$ is the reaction torque of the EFAC-BGHD annular region to the rheometer, $T_{3}$ is the CDKJ torque in the top region, $T_{4}$ is the ABON torque in the bottom region, $T_{5}$ is the torque received by the bottom annular region of the FCJI-DHLK region, and $T_{6}$ is the torque received by the top MNAE-OPBG region.

Since the tangential velocity of the rotation axis is zero, the tangential velocities of the blades AC and BD are $V = \omega r$, where $\omega$($\omega = 2\pi n$) represents the angular velocity of the blade rotation. The corresponding shear rate is defined as

28 $$\dot{\gamma } = \frac{V}{r} = \frac{\omega D/2}{{D/2}} = \omega .$$

#### Torque of regional ABCD

The torque of the blade AB and CD is

29 $$T_{1} = \tau dh\pi r = \frac{{\pi D^{2} h}}{2}\left( {\tau_{y} + k\omega^{n} } \right)$$

Previous studies^39–41^ have shown that no shear behavior occurs in the ABCD region when the blade is rotating. When the blade rotates, the surface of the blade exerts a tangential force along the surface direction on the concrete in the ABCD region. The centrifugal force generated by the rotating fluid in the ABDC region is completely balanced by the fluid outside the ABDC region, and there is no radial displacement. After the rotational speed of the rheometer shaft is stable, the rotational speed of the fluid in the ABDC region is consistent with the rotational speed of the blade, and the fluid and the blade have no relative movement trend in the radial and tangential directions. Therefore, the fluid in the ABDC region does not apply shear stress to the blade, that is, the required torque ${T}_{1}$ = 0.

#### Ring region EFAC-BGHD torque ${T}_{2}$

This region can be simplified as an annular flow, and the speed of the blade is AC/BD. The speed of the blade is the boundary speed $V = \omega r$, and the effective width of the region is $e = \left( {D_{t} - D} \right)/2$. At this time, the shear rate $\dot{\gamma }$ of the region is $V\left( e \right)$, so the corresponding torque $T_{2}$ is

30 $$\smallint dT_{2} = \left[ {\tau_{y} + k\left( \frac{V}{e} \right)^{n} } \right]\frac{{\pi D^{2} h}}{2}.$$

#### Torque $T_{3}$ of region CDKJ

This area can be simplified into two rotating plates, and the upper part of the plate rotates unilaterally, for the occurrence of concrete shear. The velocity $V$ along the radius direction on the plate is $\omega r$, so it's shear rate $\dot{\gamma }$ is

31 $$\dot{\gamma }_{CDKJ} = \frac{\omega r}{{z_{2} }}$$

The torque integral $T_{3}$ acting on the micro-element in this region is

32 $$\smallint dT_{3} = \mathop \smallint \limits_{0}^{\frac{D}{2}} \tau \cdot 2\pi r^{2} dr = \frac{{\pi D^{3} }}{12}\tau_{y} + \frac{{2\pi k\omega^{n} }}{{z_{2}^{n} }} \cdot \frac{1}{n + 3} \cdot \left( \frac{D}{2} \right)^{n + 3} .$$

#### Torque $T_{4}$ of region ABON

The force analysis of the torque in this region is similar to that of $T_{3}$, and the integral of this region is

33 $$\smallint dT_{4} = \mathop \smallint \limits_{0}^{\frac{D}{2}} \tau \cdot 2\pi r^{2} dr = \frac{{\pi D^{3} }}{12}\tau_{y} + \frac{{2\pi k\omega^{n} }}{{z_{1}^{n} }} \cdot \frac{1}{n + 3} \cdot \left( \frac{D}{2} \right)^{n + 3} .$$

#### Torque $T_{5}$ of region FCJI-DHLK

Considering that the velocity of any height z in this region is

34 $$V_{r} = \frac{z}{{z_{2} }}\omega r = \frac{Vz}{{z_{2} }}$$

The corresponding shear rate is defined by the shear rate as

35 $$\dot{\gamma }_{FCJI - DHLK} = \frac{{V_{r} }}{e} = \frac{Vz}{{z_{2} e}}$$

The torque contribute in this region is

36 $$\int {dT_{5} } = \mathop \smallint \limits_{0}^{\frac{d}{2}} \mathop \smallint \limits_{0}^{{z_{2} }} \left( {\tau_{y} + k\left( {\frac{Vz}{{z_{2} e}}} \right)^{n} } \right)2\pi rdzdr = \frac{{\pi D^{2} z_{2} }}{2}\left( {\tau_{y} + k\left( {\frac{{V_{r} }}{e}} \right)^{n} \frac{1}{n + 1}} \right).$$

#### Torque $T_{6}$ of region MNAE-OBGP

The analysis process of this region is similar to $T_{5}$, so the value is

37 $$T_{6} = \frac{{\pi D^{2} z_{1} }}{2}\left( {\tau_{y} + k\left( {\frac{{V_{r} }}{e}} \right)^{n} \frac{1}{n + 1}} \right)$$

Therefore, the overall torque Eq. (21), because during the test, the upper and lower immersion heights of the rheometer are the same, that is, $z_{1} = z_{2} = z$, which is brought into Eqs. (26), (27), (30) and (31) with $T_{2} = T_{3}$ and $T_{5} = T_{6}$, and $T_{1} = 0$. Therefore, the total torque contributed by the test area to the rheometer is

38 $$T = 2T_{2} + T_{4} + 2T_{5}$$

39 $$T = \pi D^{2} \left( {z + \frac{h}{2} + \frac{D}{6}} \right)\tau_{0} + \frac{{\pi D^{2} k}}{2}\left[ {h\left( \frac{D}{2e} \right)^{n} + \frac{2Z}{{n + 1}}\left( \frac{D}{2e} \right)^{n} + \frac{2}{{\left( {n + 3} \right)Z^{n} }} \cdot \left( \frac{D}{2} \right)^{n + 1} } \right]\left( {2\pi N} \right)^{n}$$

When $n$ = 1, the fitting method of Eq. (33) corresponds to the Bingham model, which simplifies the relationship between torque and speed, that is,

40 $$T = \pi D^{2} \left( {z + \frac{h}{2} + \frac{D}{6}} \right)\tau_{0} + \frac{{\pi D^{2} k}}{2} \cdot \left[ {\frac{hD}{{2e}} + \frac{ZD}{{2e}} + \frac{1}{2Z} \cdot \left( \frac{D}{2} \right)^{2} } \right] \cdot 2\pi N$$

41 $$\frac{T}{{\pi D^{2} \left( {z + \frac{h}{2} + \frac{D}{6}} \right)}} = \tau_{0} + \frac{{\frac{{\pi D^{2} k}}{2} \cdot \left[ {\frac{hD}{{2e}} + \frac{ZD}{{2e}} + \frac{1}{2Z} \cdot \left( \frac{D}{2} \right)^{2} } \right]}}{{\pi D^{2} \left( {z + \frac{h}{2} + \frac{D}{6}} \right)}} \cdot 2\pi N$$

The calculation result of Eq. (34) is the same as the Bingham fitting formula deduced by Laskar et al^42^, which indirectly proves the correctness of the derivation form. In the actual measurement process of the rheometer, it is not a linear relationship similar to the Bingham form, but a nonlinear Bi-viscosity form Eq. (33) is written as

42 $$\tau = T/\pi D^{2} \left( {z + \frac{h}{2} + \frac{D}{6}} \right) = \tau_{0} + K\left( n \right)N^{n}$$

Substituting the geometric parameters of the rheometer blade $h$ = 0.127 m, $z$ = 0.114 m, D = 0.127 m, $D_{t}$ = 0.38 m to Eq. (36), the corresponding relationship between shear stress and torque can be obtained :

43 $$\tau = \frac{T}{{\pi D^{3} \left( {\frac{{\left( {h + 2z} \right)}}{D} + \frac{1}{3}} \right)}} = 199.0551T$$

The shear strain corresponding to rotation is

44 $$\dot{\gamma } = 2\pi N = 6.2832N$$

Similarly, the shear rate of the Bingham model is

45 $$\dot{\gamma }_{B} = \frac{{\pi D^{2} }}{2}*\frac{{\left[ {\frac{hD}{{2e}} + \frac{ZD}{{2e}} + \frac{1}{2Z} \cdot \left( \frac{D}{2} \right)^{2} } \right]}}{{\pi D^{3} \left( {\frac{{\left( {h + 2z} \right)}}{D} + \frac{1}{3}} \right)}}*2\pi N = 4.3938N.$$

## Experimental study

### Experimental material

**Figure 6:**
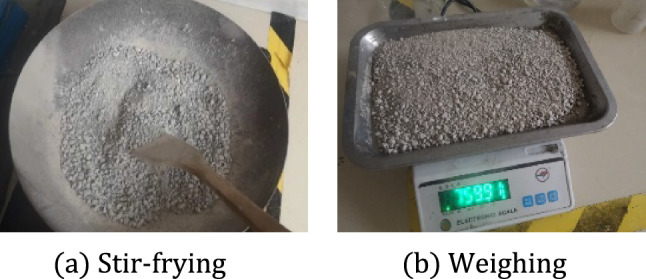
Moisture determination.

The materials used in the four groups of concrete experiments for this test are ordinary Portland cement PO42.5, with a density of 3050 ${\text{kg}}/{\text{m}}^{3}$. The type of fly ash is F class two, and the density is 2240 ${\text{kg}}/{\text{m}}^{3}$. The type of ore powder is S95, and the density is 2880 ${\text{kg}}/{\text{m}}^{3}$. The type of coarse aggregate is natural pebble, and its continuous gradation is 4.75–20 mm. The average aggregate density measured by multiple drainage method is 2600 ${\text{kg}}/{\text{m}}^{3}$. The fine aggregate is machine-made sand and stone, and the humidity is 3.5%. The measurement method is shown in Fig. 6. The additive is a high-efficiency retarding water reducer, and the parameter is 9%

In order to comprehensively study the influence of the rheological properties of concrete on the flow process, different proportions of concrete were prepared in this paper to prevent contingencies during the experiment. The specific match is shown in Table 1.

**Table 1:** Experimental group concrete ratio (${\text{kg}}/{\text{m}}^{3}$).

Group	Coarse	Sand	Water	Cement	Fly ash	Mineral powder	Additive
E1	1069.8	1133.6	175.3	258.0	58.7	82.1	8.8
E2	1113.6	1089.0	176.1	258.0	58.7	82.1	8.8
E3	1026.2	1185.1	167.5	258.0	58.7	82.1	8.8
E4	982.6	1229.7	166.4	258.0	58.7	82.1	8.8

**Figure 7:**
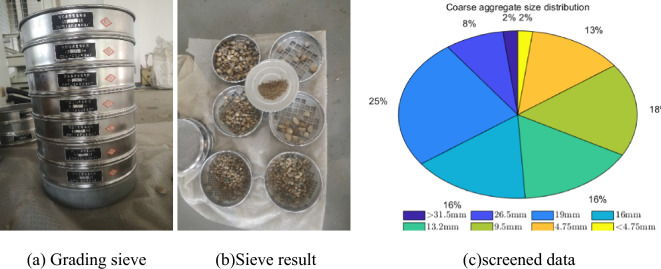
Grading of aggregates.

Considering that the material used in the experimental process is less, it is necessary to accurately control the quality of the material, especially the control of the water content has a great influence on the experimental results. In addition, in order to simulate the movement of large aggregate in concrete, the size and gradation distribution of aggregate in concrete are screened as shown in Fig. 7.

### Slump test

**Figure 8:**
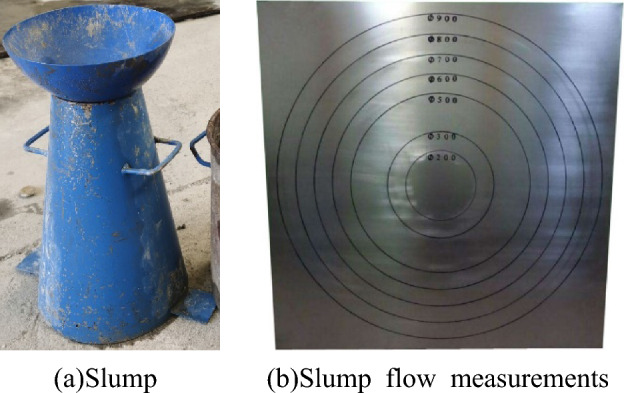
Slump experimental instrument.

The slump test is to detect the pumpability of concrete. Generally, the fluidity of concrete with slump greater than 180 mm is higher. The specific structure of the slump instrument is a cone with a top diameter of 100 mm, a bottom diameter of 200 mm, and a height of 300 mm, as shown in Fig. 8a and b.

**Figure 9:**
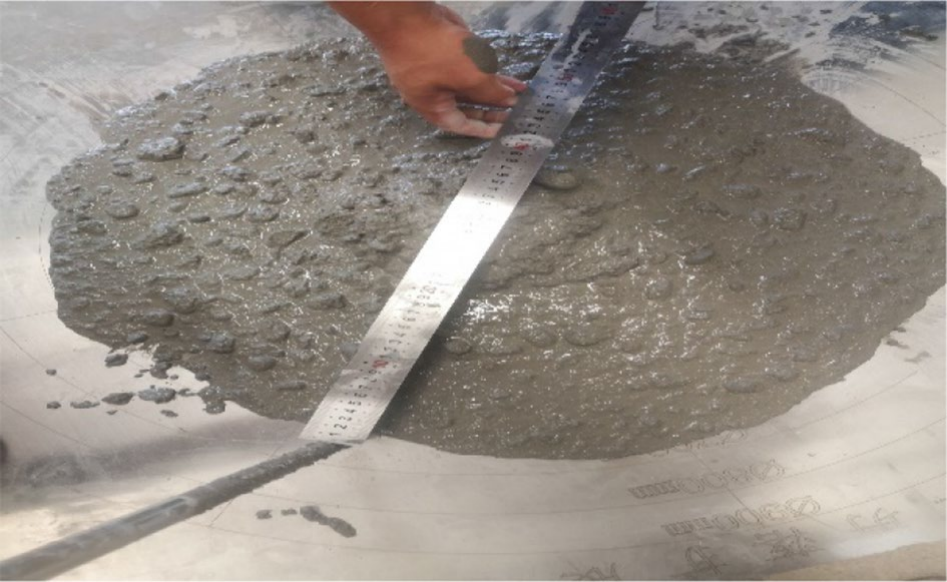
Slump expansion test.

In order to ensure the relative stability of the data obtained during the collapse process, the above four experimental groups were stirred and tested as shown in Fig. 9, and the average value of the slump s and the two-direction expansion $s_{f}$ was taken. The average value of the two extensions is $s_{af}$, and the experimental results are shown in Table 2.

**Table 2:** Experiment result.

Group	$s_{f1}$	$s_{f2}$	$s$	$s_{af}$
E1	580	600	235	590
E2	460	460	225	460
E3	470	480	235	475
E4	490	460	225	475

### Concrete rheological test

**Figure 10:**
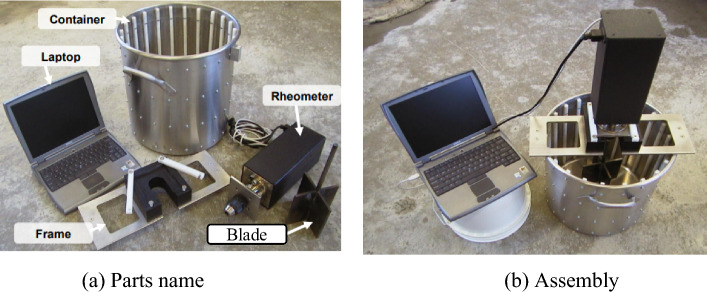
Concrete rheometer.

The torque of the rheometer blade during rotation is calculated to convert the rheological properties of the concrete. ICAR Rheometer is used, as shown in Fig. 10. During the test process, the rheometer is standing still, and the rheometer is sunk into the fresh concrete by gravity. When the rheometer is started, the initial speed of the blade will change to 0.55rev/s. After a period of operation, the speed will decrease by 0.075rev/s. After multiple decelerations, the minimum measurement speed is 0.15rev/s, and the torque value T of the rheometer at multiple test sampling points is recorded.

**Figure 11:**
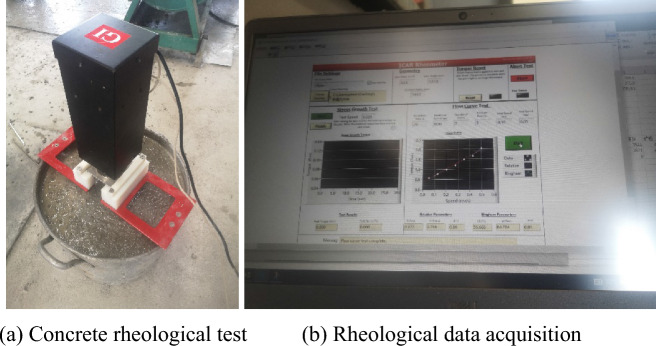
Rheological test of fresh concrete.

The rheometer in Fig. 10 is divided into several parts, which are laptop, rheometer, blade, support frame and container cylinder. The rheometer is assembled when used, and the blade is inserted into the fresh concrete as shown in Fig. 11a. The torque data of the rotation process is read and recorded, and the recording process is shown in Fig. 11b.

**Figure 12:**
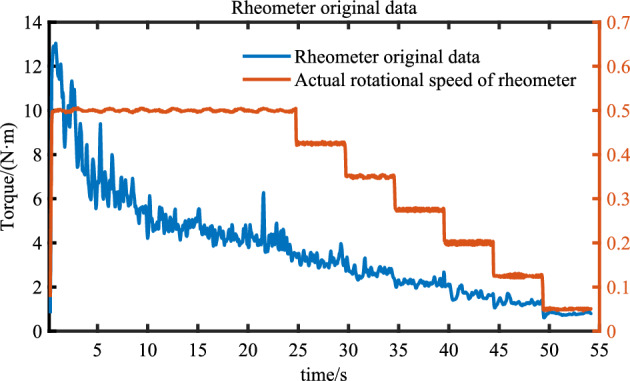
Original data.

Considering that during the operation of the rheometer, if the blade stirs to a larger aggregate, a certain fluctuation will occur, which will affect the final calculation results and thus affect the analysis results. Therefore, it is necessary to process the data of the rheometer. The original data of the rheometer is shown in Fig. 12.

**Figure 13:**
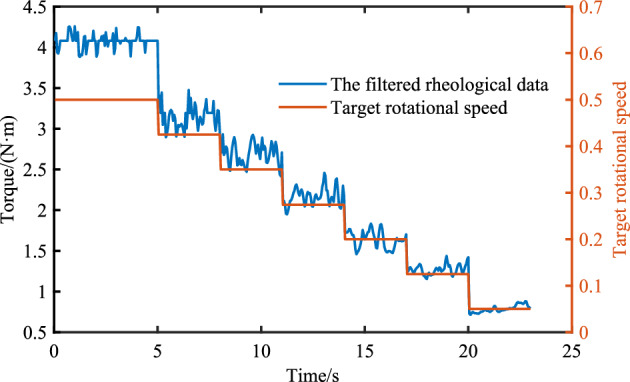
Filtered data.

Obviously, the data in Fig. 12 fluctuate greatly when the rotational speed of the rheometer changes or the larger aggregate is touched. The processing method of the original data is to select the last 3 s after the rotational speed of the rheometer is stable as the torque at the current rotational speed. In addition, it is also necessary to filter out the data points with large fluctuations in the rotation process, and then take the average torque in the speed range. The torque distribution after filtering is shown in Fig. 13.

The rheological test data of the concrete in the experimental group are shown in Table 3. Substituting the data in the table into Eqs. (36), (38) and (39), the Bingham and Bi-viscosity model data corresponding to the experimental group can be obtained, as shown in Table 4.

**Table 3:** Relationship between torque and rotational speed in experimental group.

Rotation rate (r/s)	E1 (${\text{N}} \cdot {\text{m}}$)	E2(${\text{N}} \cdot {\text{m}}$)	E3(${\text{N}} \cdot {\text{m}}$)	E4(${\text{N}} \cdot {\text{m}}$)
0.05	0.323	0.537	0.492	0.523
0.125	0.515	0.876	0.778	0.709
0.2	0.776	1.148	1.065	0.919
0.275	0.874	1.485	1.151	1.045
0.35	0.998	1.745	1.397	1.208
0.425	1.246	2.083	1.672	1.425
0.5	1.517	2.326	2.044	1.559

**Table 4:** Relationship between shear stress and shear rate.

$\dot{\gamma }$($1/{\text{s}}$)	$\dot{\gamma }_{B} \left( {1/{\text{s}}} \right)$	$\tau_{E1}$(Pa)	$\tau_{E2}$(Pa)	$\tau_{E3}$(Pa)	$\tau_{E4}$(Pa)
0.314	0.22	104.10	97.94	64.29	106.90
0.785	0.55	141.13	154.86	102.51	174.37
1.256	0.88	182.93	211.99	154.47	228.52
1.73	1.21	208.01	229.11	173.97	295.60
2.20	1.54	240.46	278.08	198.66	347.35
2.67	1.87	283.65	332.82	248.02	414.63
3.14	2.20	310.33	406.87	301.97	463.00

**Figure 14:**
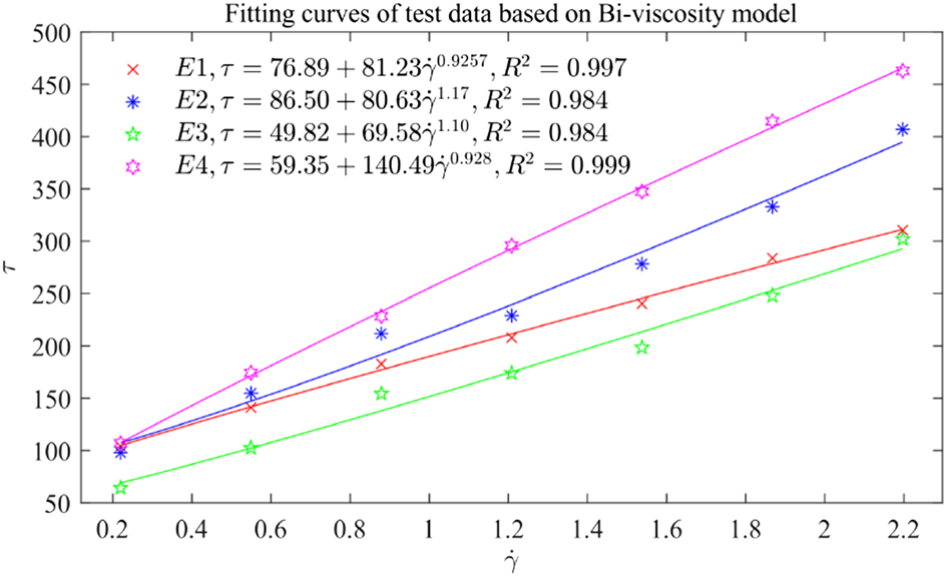
Fitting curves of test data based on Bi-viscosity model.

The data in Table 4 are substituted into the Eqs. (22), (23), (24), (26), (27), and the Bi-viscosity model of the experimental group is fitted as Table 5 and Fig. 14 by using Matlab data fitting tool.

**Table 5:** Rheological parameters of fresh concrete based on Bi-viscosity model.

Experiment group	$\tau_{y} \left( {{\text{Pa}}} \right)$	$K$(Pa·${\text{s}}^{n}$)	$n$
E1	76.89	81.23	0.9257
E2	86.50	80.63	1.17
E3	49.83	69.58	1.10
E4	59.35	140.49	0.928

**Figure 15:**
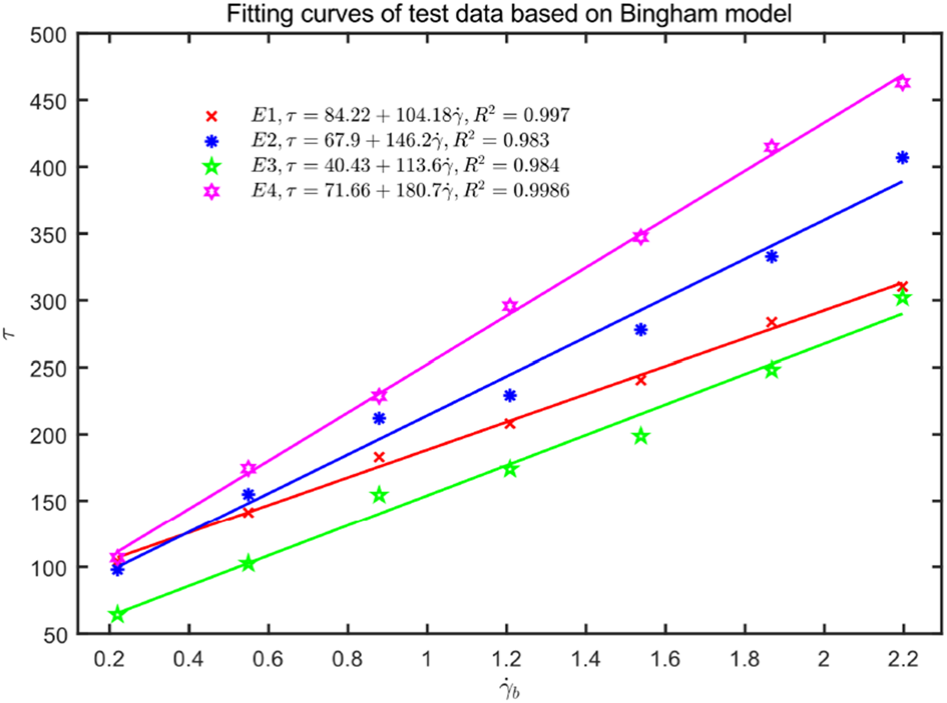
Fitting curves of test data based on Bingham model.

Similarly, the Bingham model fitting can be obtained as shown in Table 6, and the corresponding Matlab data fitting is shown in Fig. 15.

**Table 6:** Rheological parameters of fresh concrete based on Bingham model.

Experiment group	$\tau_{y} \left( {{\text{Pa}}} \right)$	$\mu_{p}$ (Pa·s)
E1	84.22	104.18
E2	67.9	146.2
E3	40.43	113.6
E4	71.66	180.7

## Simulation analysis of rheological behavior of concrete

The solver used for this work is the explicit integration solver Symplectic. The fluid–solid interaction part uses Inlet/outlet boundaries with an initial time step of 1e−6. The computation time step is automatically adjusted based on the CFL number. The GPU of the computer is NVIDIA RTX A60000, and the CPU has 96 cores.

### Verification of fluid–solid interaction (FSI)

**Figure 16:**
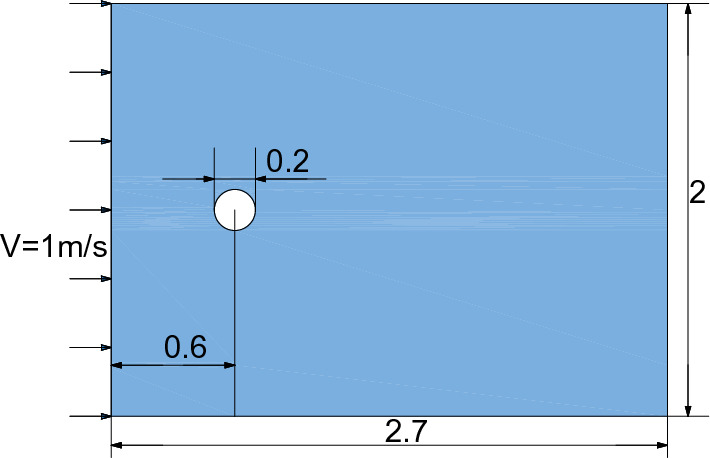
Geometry used for the analysis based on drag coefficient.

In order to obtain the accuracy of the fluid force on the solid during the SPH-DEM coupling process, a simple cylindrical flow model is established. As shown in Fig. 16, the fluid drag coefficient $C_{d} = \frac{Fd}{{\frac{1}{2}\rho V^{2} d}}$ of the cylinder is calculated at different Reynolds numbers $Re = \rho Vd/\mu$.

**Figure 17:**
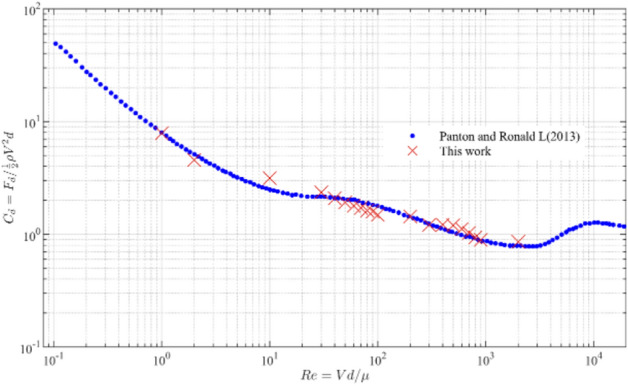
Drag coefficient $C_{d}$ as a function of the Reynolds number ${\text{Re}}$.

At different conditions of Reynolds number $\left( {Re} \right)$, the calculated drag coefficient is compared with the experimental data of Panton et al^43^. The data are shown in Fig. 17.

The data in Fig. 17 show that under the condition of low Reynolds number $\left( {Re < 2000} \right)$ the calculation results of the resistance coefficient are in good agreement with the experimental results. Therefore, for the fluid–solid coupling problem under laminar flow conditions, the SPH-DEM method can be well characterized. This method is now applied to the study of fresh concrete.

### Simulation analysis model of concrete

**Figure 18:**
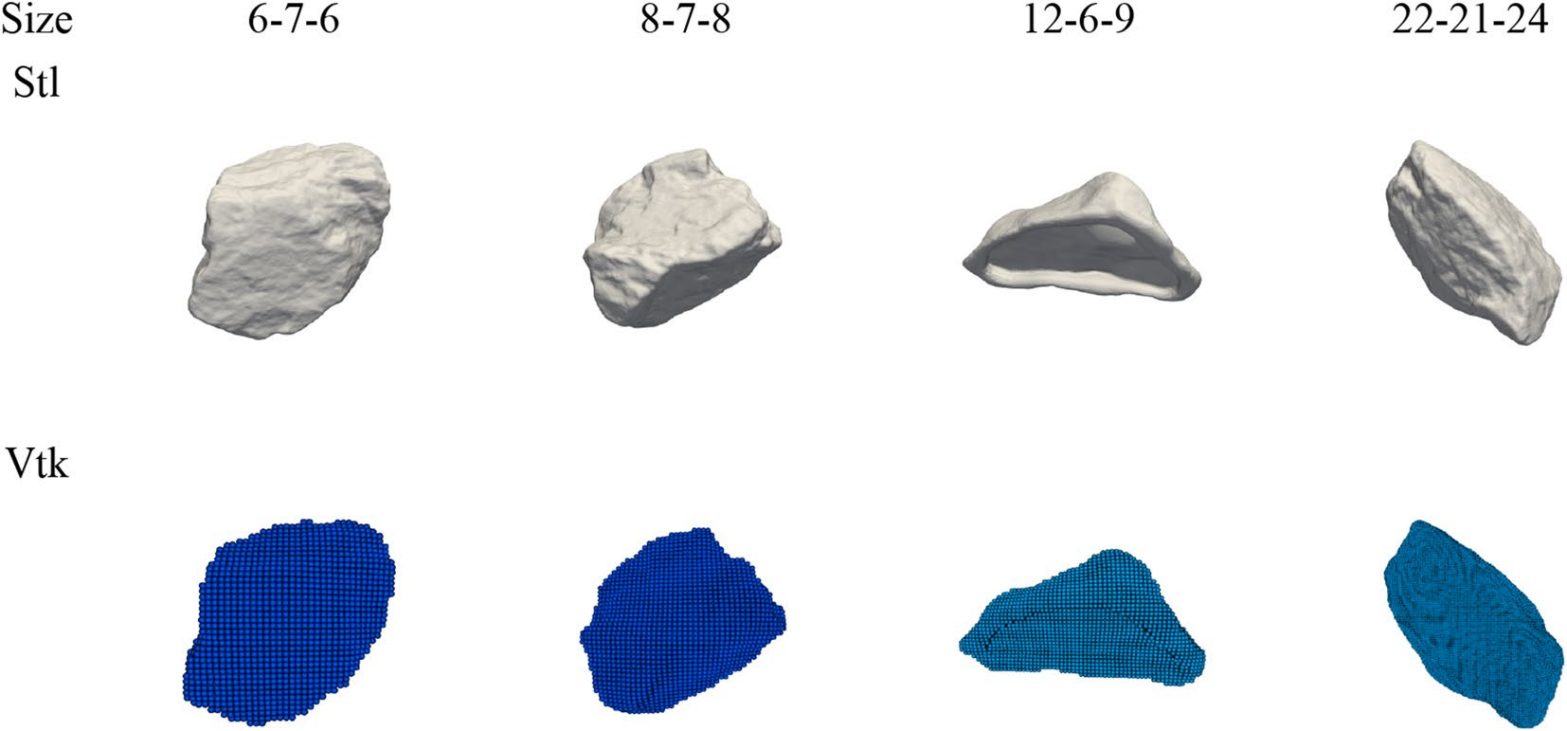
Coarse aggregate and point lattices.

The simulation model of concrete slump is composed of two phases, which are slurry, generated by fluid particles of Bi-viscosity model, and large aggregate DEM solid. The aggregate file is scanned into stl format and converted into SPH particles to form rigid large particle Vtk format, as shown in Fig. 18. The numbers in the figure is the size of the aggregate (x–y–z) in three directions (mm).

**Figure 19:**
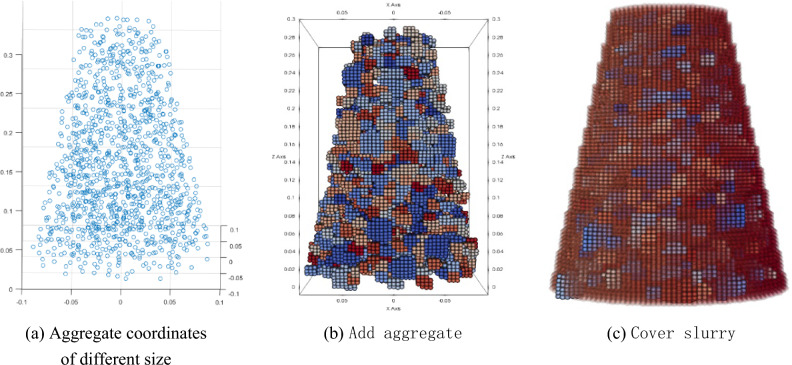
Concrete simulation model generation.

The concrete slump instrument is filled with slurry and coarse aggregate, and the proportion of slurry and aggregate is calculated by the results given in Fig. 7. Since the position of the aggregate in slump test is random, the aggregate initial position in the simulation model also needs to be randomly generated. Here, the Matlab Monte Carlo random method generates aggregate positions of various sizes in the slump instrument, as shown in Fig. 19. Simulation parameters shown in Table 7.

**Table 7:** Simulation parameters.

Type	Parameter	Notation	Units	Value
Nature pebbles	Young modulus	$E$	GPa	30
	Poisson ratio	$\nu$		0.35
	Restitution coefficient	$r$		0.45
	Kinetic friction efficient	$\mu$		0.35
	Density	$\rho$	kg/m^3^	2600
Simulation parameters	CFL coefficient	$C_{FL}$		0.2
	Initial time interval	${\Delta }t$	s	1e−6
	The artificial viscosity coefficient	$\alpha_{II} ,\beta_{II}$		0.001
	Sound speed coefficient	$\beta$		20
	Constant of EOS	$\gamma$		7
	Particle density	$\rho_{f}$	kg/m^3^	2100
	Simulation time	$t$	s	5

### Simulation analysis of concrete slump

**Figure 20:**
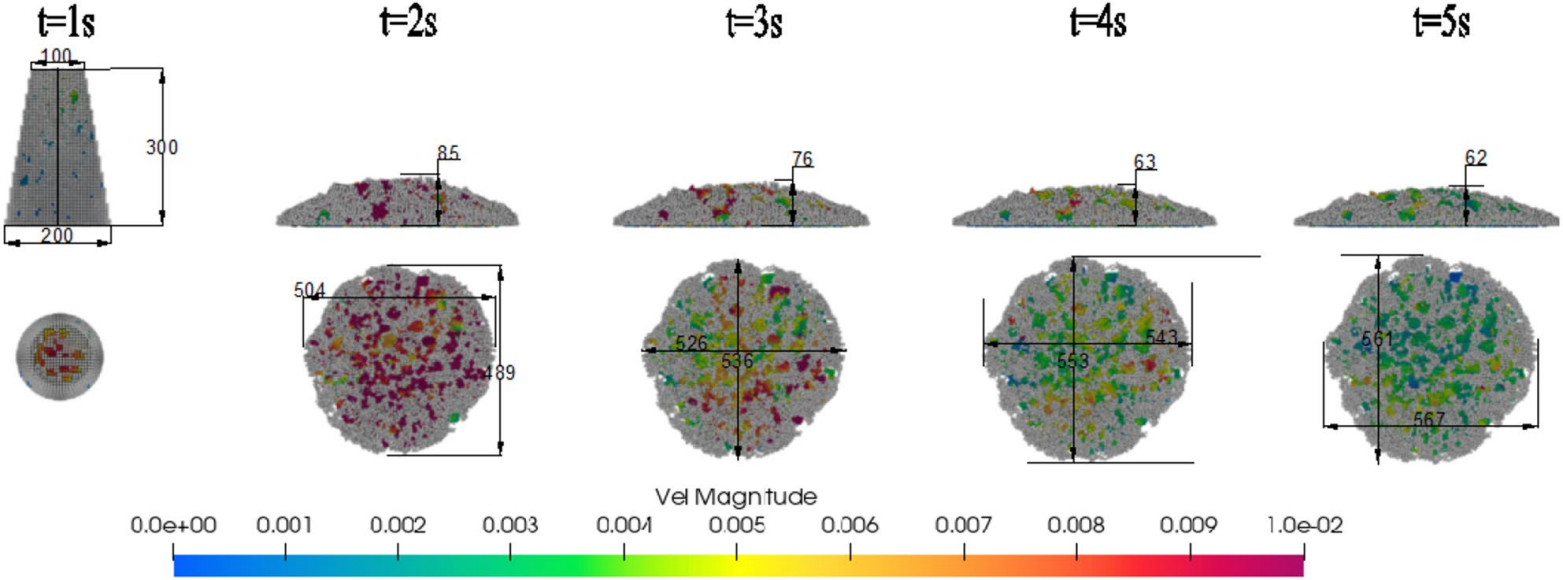
Simulation result of first group $E_{1}$.

**Figure 21:**
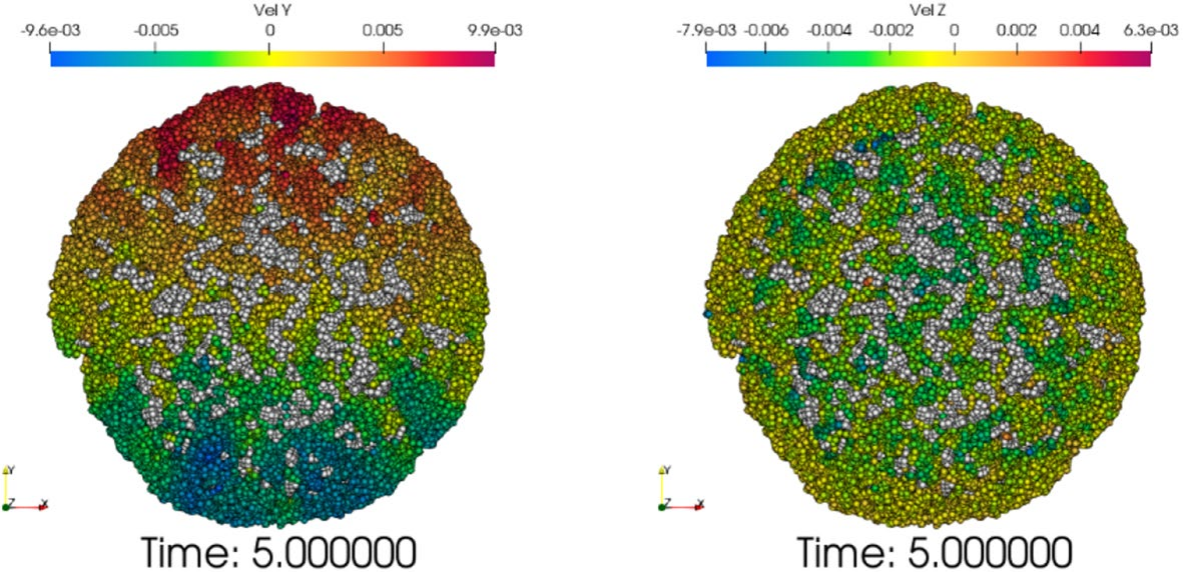
Slurry velocity in Y and Z directions.

After the concrete slump model is established, the rheological parameters and aggregate friction coefficient of the experimental group are substituted for simulation analysis. The simulation and experimental results are compared. The first set of simulation analysis is shown in Fig. 20. In order to facilitate the distinction between coarse aggregate and slurry, slurry in Fig. 20 is represented by gray, and the other is shown by velocity legend. When the coarse aggregate stops moving, the slump height will hardly change at this time. Slurry velocity could of slurry in 5 s is shown in Fig. 21.

**Figure 22:**
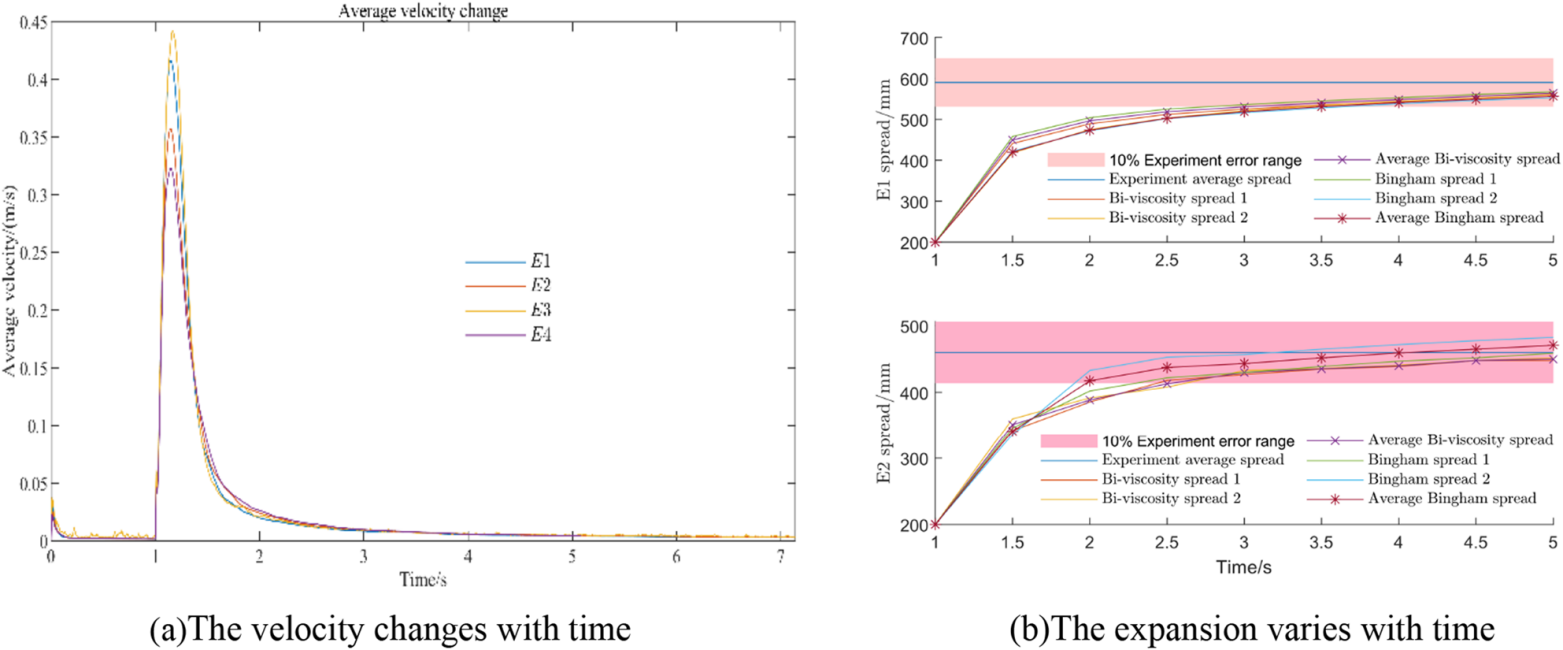
The velocity and spread change with time.

It can be seen from Fig. 22a that at 1 s, the movement speed of the lifting cylinder and the concrete will gradually decrease, from the initial high-speed movement, a sharp decline to a stable process. The simulation slump is 63 mm at 4 s, and only 1 mm at 5 s. Compared with the difference of 13 mm between 3 and 4 s, the change is very small, so the simulation time is 5 s. The simulation data and experimental data results are shown in Fig. 21b. It can be seen from the diagram that compared with the experimental group $E_{1}$, $E_{2}$, it can be seen that the Bi-viscosity model can achieve a corresponding high accuracy range similar to the Bingham model. Compared with the experimental values, the error ratio is less than 10%. Combined with the slump simulation results of Fig. 22b, it can be seen that the overall error ratio of the Bi-viscosity model does not exceed 10%.

**Figure 23:**
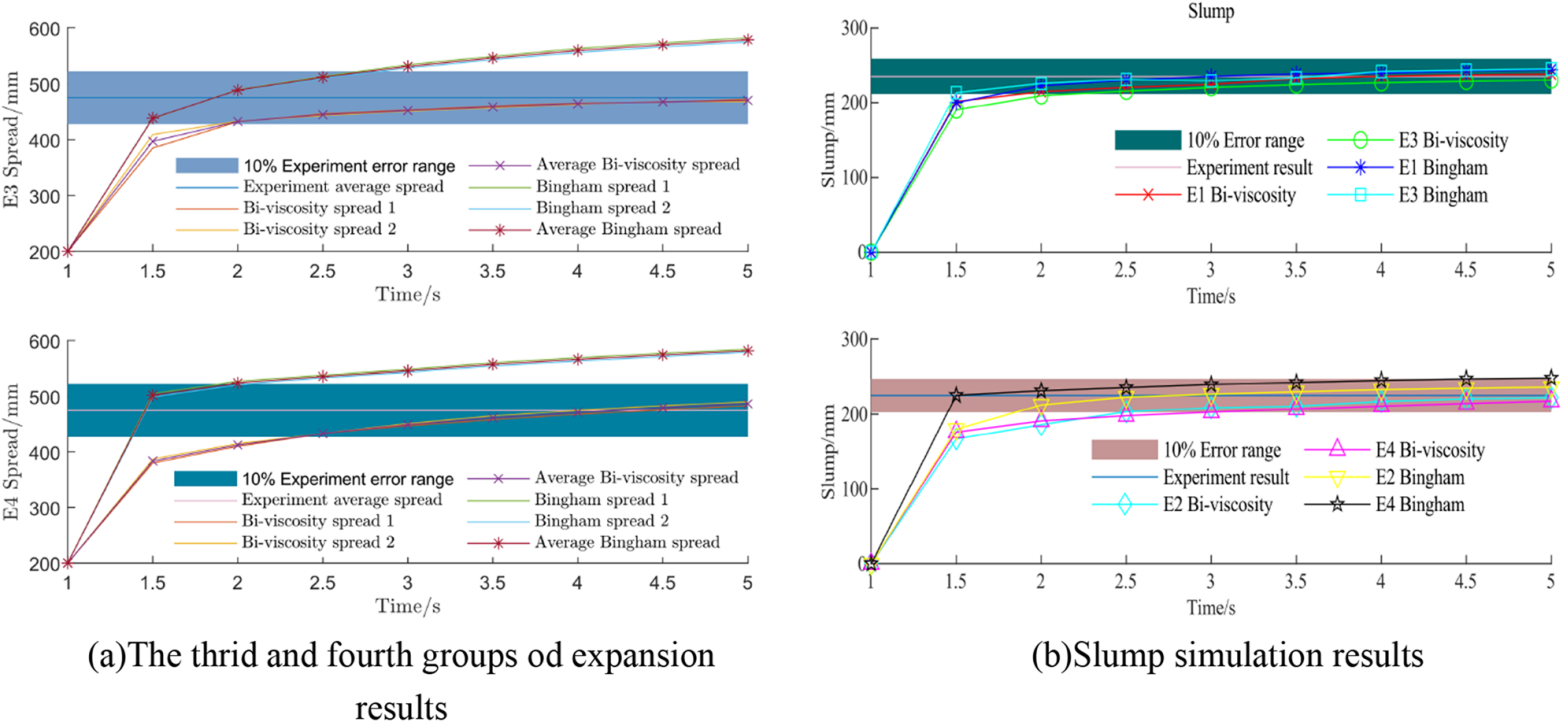
Slump and expansion simulation results.

The results of the third $E_{3}$ and fourth $E_{4}$ experimental groups are shown in Fig. 23a and b. Compared with the experimental groups $E_{3}$ and $E_{4}$, in the case of low-speed motion, due to the characteristics of the Bingham model itself, a large yield stress is required to make the concrete easy to flow. Therefore, in this case, the Bingham model fitted by the HBP model has poor numerical fitting accuracy for the experimental group, and the relative error can reach 20%, while the Bi-viscosity model still has high fitting accuracy for the rheological experiment, and its error percentage can be less than 10%.

### The influence of rheological parameters on the slump results

#### Effect of yield stress on slump

**Figure 24:**
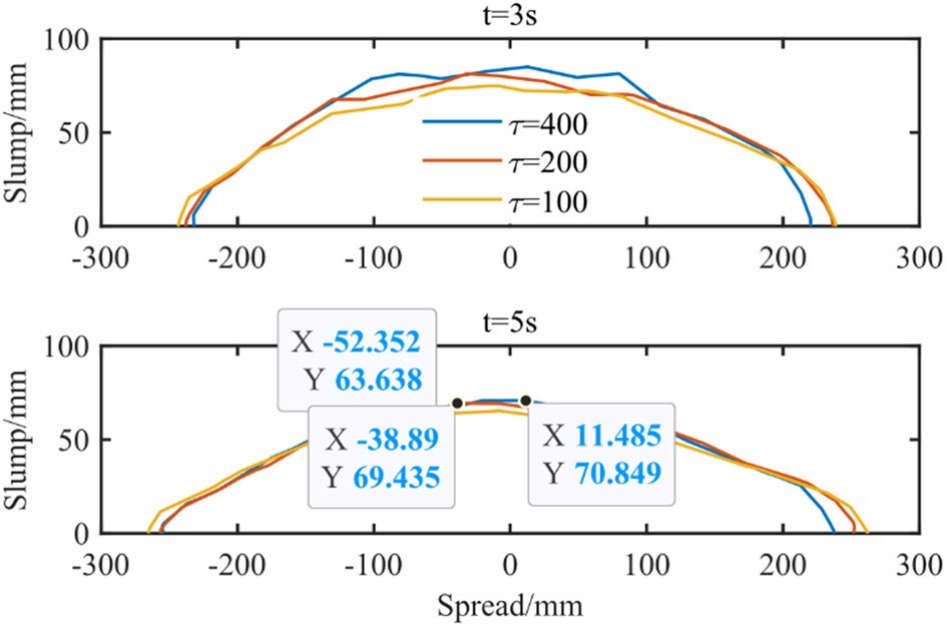
Effect of yield stress on slump test.

There are three main parameters in the Bi-Viscosity model, which will affect the simulation results. Based on the Bi-viscosity rheological parameters of the experimental group $E_{1}$ the yield stress in the model was changed to simulate. Considering the Bingham model used in the empirical formula $s = 255 - 176\tau /\rho$^44^, *n* takes 1 here for comparative analysis. Considering that the yield stress is related to the anti-deformation ability of the fluid, the greater the yield stress, the stronger the anti-deformation ability, and the slower the flow. The simulation analysis results are shown in Fig. 24. It can be seen that as the yield stress increases.

Comparing the simulation results with the empirical formula, it can be obtained that the simulated slumps with yield stress of 400, 200 and 100 are 230 mm, 231 mm and 237 mm, respectively, while the empirical formula results are 246 mm, 237 mm and 220 mm. Therefore, the error percentages of simulation analysis and empirical formula are 6.6%, 2.8% and 7.5%, respectively. The overall error percentage is less than 10%.

#### The influence of coarse aggregate shape on simulation results

**Figure 25:**
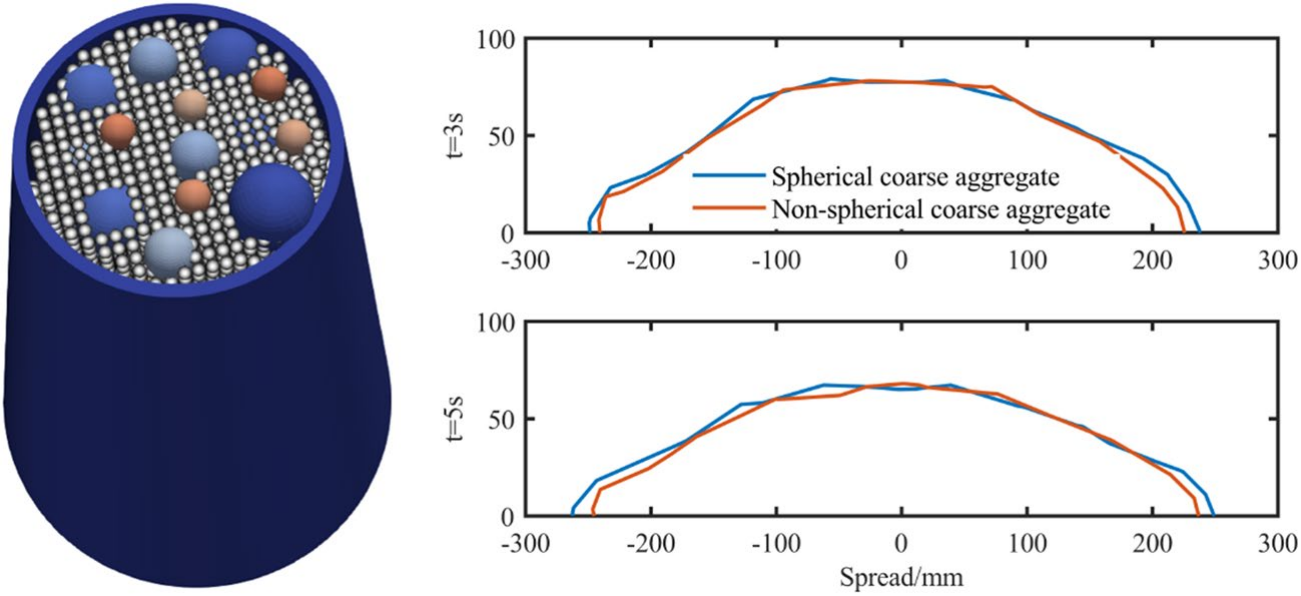
Comparison of spherical and non-spherical aggregate.

The comparison between spherical coarse aggregate and non-spherical coarse aggregate is mainly to consider the interaction between aggregate shape and bottom and aggregate. Because the contact and action range of spherical coarse aggregate is less than that of non-spherical coarse aggregate. Therefore, the expansion range of spherical coarse aggregate should be larger than that of non-spherical aggregate. The simulation results are shown in Fig. 25. The results of Fig. 25 are consistent with the trend of theoretical analysis results.

#### The influence of power law coefficient on the simulation analysis of slump

**Figure 26:**
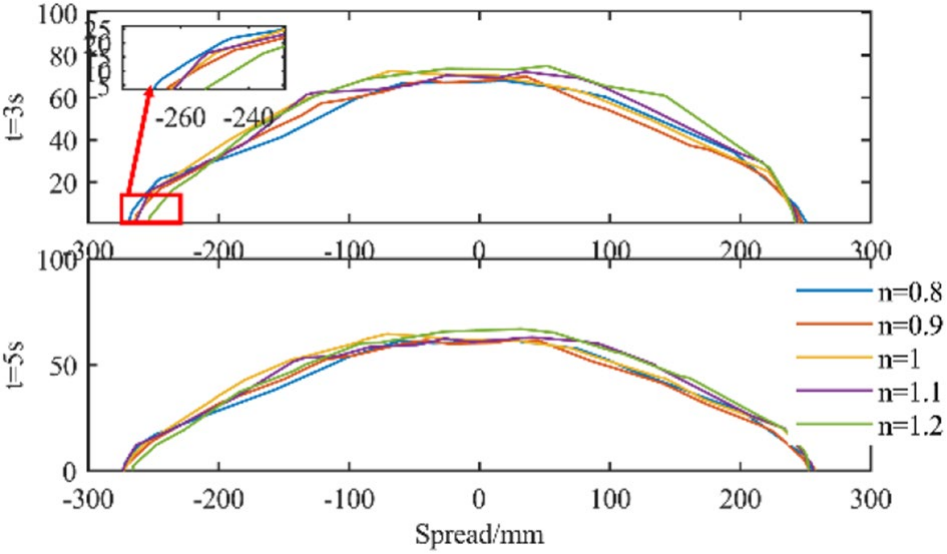
Influence of power-law coefficient on slump analysis.

The power law coefficient in the Bi-viscosity model is changed to explore the influence of its change trend on the concrete slump experiment, as shown in Fig. 26. It can be seen that when the initial acceleration changes greatly, the expansion range of the shear thinning fluid is obviously larger than that of the shear thickening fluid. With the extension of the simulation time, when the overall acceleration changes little, the corresponding shear stress is similar to the yield stress. Therefore, for the final result, it tends to the expansion of n = 1, and the overall impact is not obvious.

#### The influence of power law coefficient on the simulation analysis of slump

**Figure 27:**
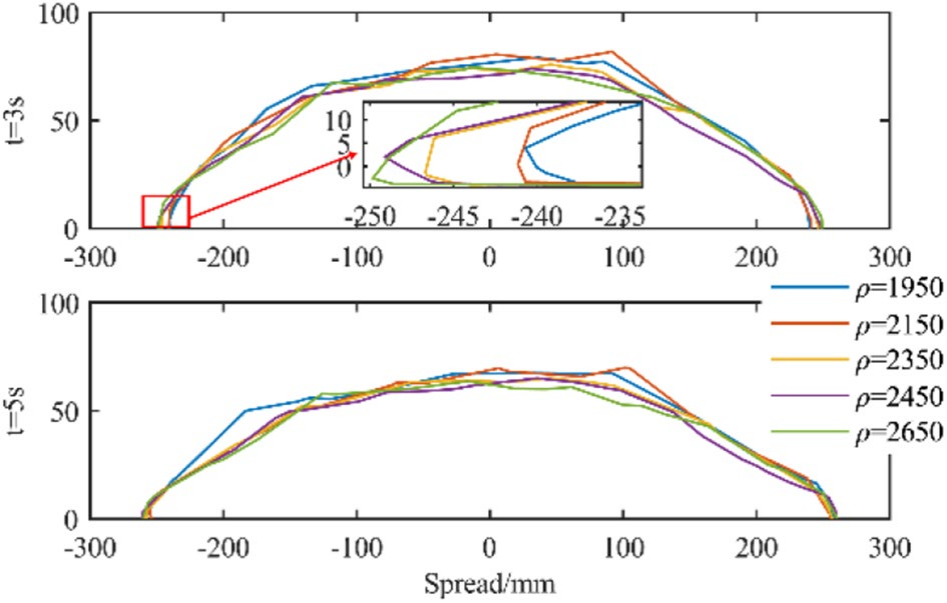
Influence of fluid density on slump.

Changing the fluid density for slump simulation analysis, the most intuitive change brought by density is the difference in gravity, which leads to the difference in shear stress corresponding to the motion process. The greater the density, the greater the shear stress, and vice versa. Therefore, the slump and expansion of the fluid with higher density will increase, and the slump of the fluid with lower density is relatively small. The simulation results are shown in Fig. 27.

## Comparison of simulation and experimental results of concrete pumping process

**Figure 28:**
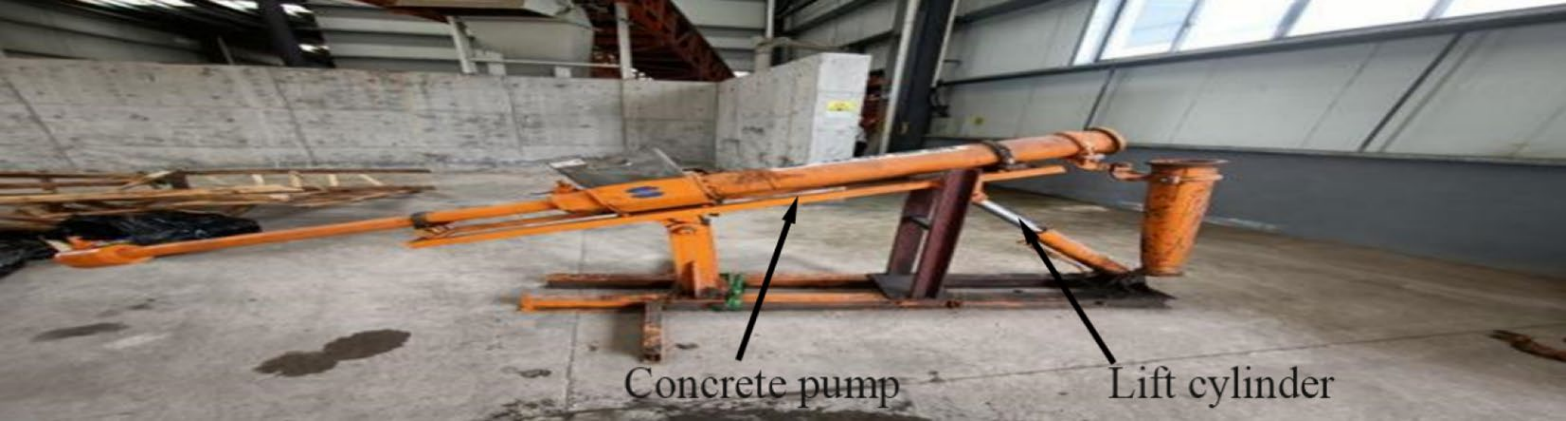
Pumping platform.

The industrial application of fresh concrete is mainly concrete pumping. In order to explore the applicability of the model in industrial application, a simple pumping platform is established, as shown in Fig. 28. The pumping pressure of the experimental group is compared with the pressure change in the simulation analysis of the pumping push process. If the matching degree is high, it can provide some solutions and methods for the coarse aggregate motion trajectory, pipe plugging reason, pressure prediction and analysis in the concrete pumping process.

**Figure 29:**
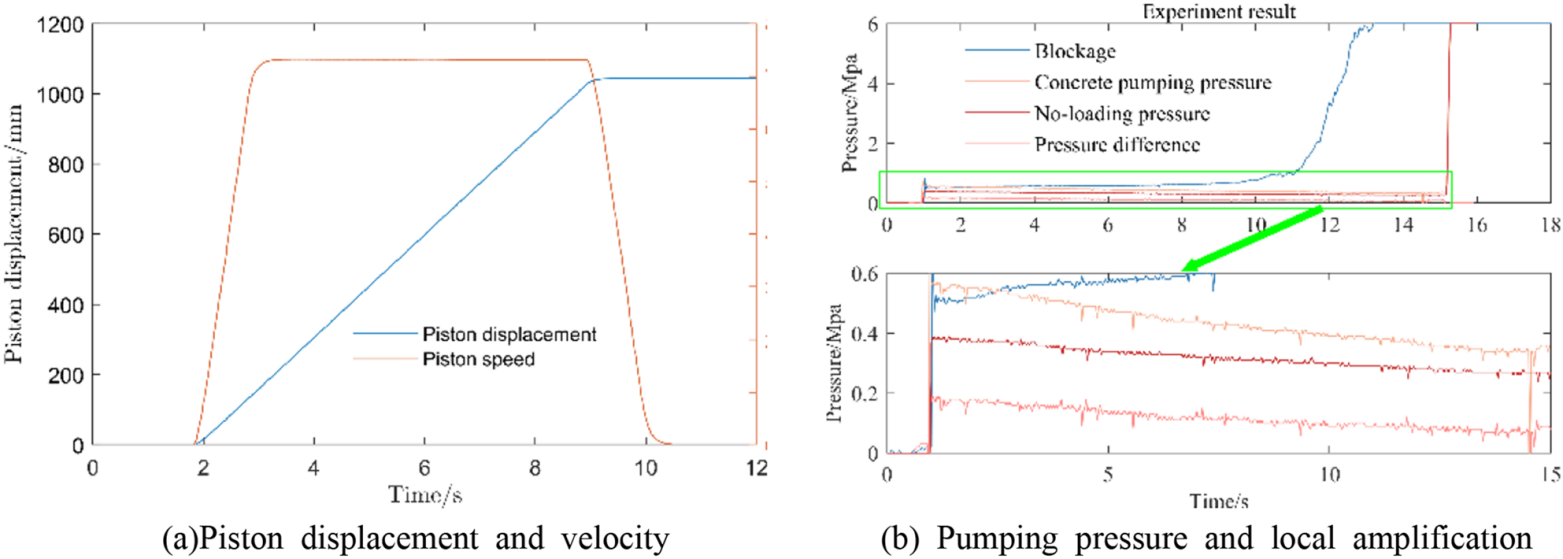
Concrete pumping experimental results.

In the initial pumping process of the concrete cylinder, in order to reduce the influence of friction resistance on the pumping pressure collection, the concrete cylinder wall is smeared with grease, and the no-load pressure after smearing grease is collected. The total pressure minus the no-load pressure of the concrete cylinder is the pressure required for the concrete pumping process. The pumping speed of the concrete cylinder hydraulic system is set to 73.6 mm/s, and the piston motion stroke is 1.04 m, as shown in Fig. 29. Under this initial condition, the pumping pressure test of the first experimental group E1 was carried out, and the test results are shown in the figure. Normal concrete shows a slight downward trend in the process of movement. According to Newton’s law of motion, when the initial velocity of concrete rises from 0 to 73.6 mm/s, the acceleration required is the largest, that is, the external force is the largest. When the velocity of concrete is relatively stable, the pressure will gradually decrease. The experimental results are consistent with the theoretical analysis.

**Figure 30:**
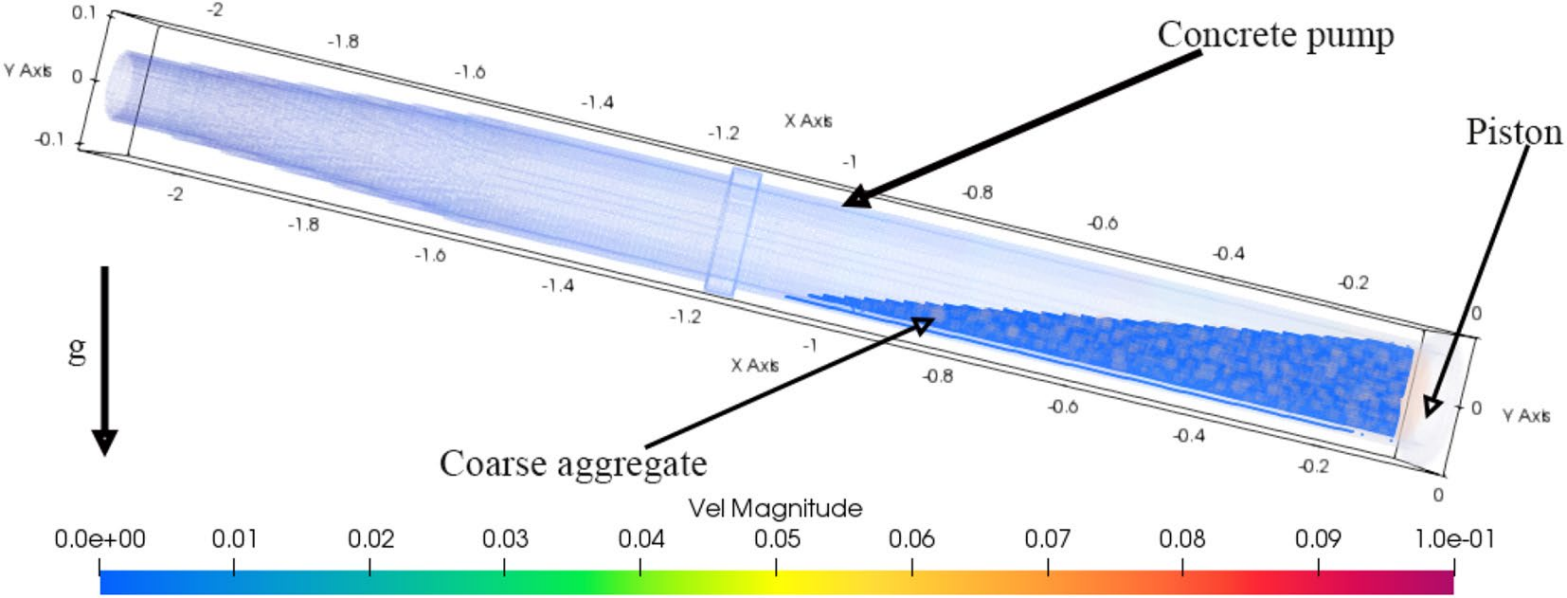
Simulation Pumping platform.

Under the above conditions, the rheological parameters are simulated and analyzed. The Bi-viscosity model $E_{1}$ group is used. The initial state is shown in Fig. 30. In the figure, the piston movement speed is 73.6 mm/s of the experimental group’s movement process, and the load is carried out by relying on the gravity of the concrete. The piston force is converted to the hydraulic system pressure and the test process is compared and analyzed.

**Figure 31:**
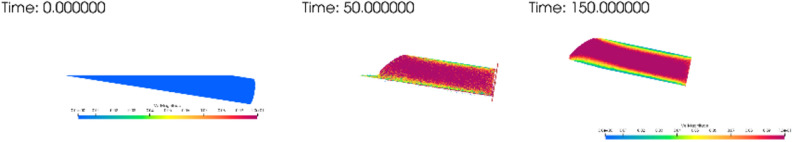
Simulation result (units = 0.1 s).

**Figure 32:**
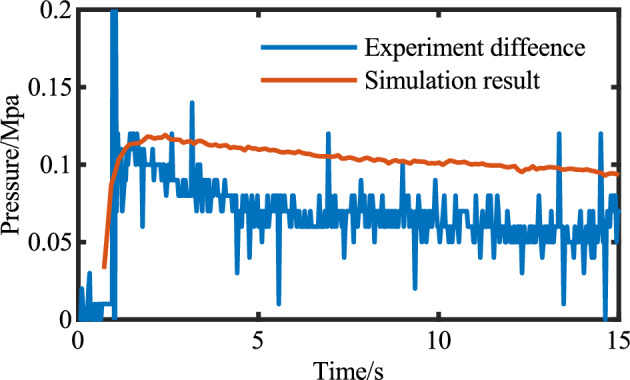
Comparsion of the simulation and experiment result.

The simulation results of group E1 are shown in Figs. 31 and 32. The simulation values are keeping same tendency with experiment in Fig. 32. Considering the movement process of concrete in the cylinder, particle migration will occur. As shown in Fig. 31, particles will move to the central high-speed area, while the boundary is formed by thin slurry. So moving resistance will be smaller.

The pressure drop trend of the motion process is higher than that of the simulation, but Fig. 32 still shows the same downward trend, and the pressure check of the initial motion state of the concrete has higher accuracy.

## Discussion and conclusion

This paper intends to use a new method to describe the rheological behavior and particle movement of concrete during pumping. The following conclusions are obtained by combining simulation and experimental results.

1. SPH-DEM fluid–solid coupling force analysis model is established, and the drag coefficient under different Reynolds numbers is calculated. The simulation results are very close to the experimental results.
2. SPH-DEM method was used to simulate the rheological behavior of fresh concrete with large particle size. The Bi-viscosity model and Bingham model of concrete were established. The slump simulation analysis model was compared with the experiment. The results show that the Bi-viscosity model has higher fitting accuracy than the Bingham model and the experiment, and the overall error is less than 10%. The fitting error of the Bingham model for the experiment is about 20%.
3. Influence of different rheological parameters of Bi-viscosity model on the simulation process of concrete slump is analyzed. The results show that the greater the critical yield stress, the stronger the anti-deformation ability of concrete, and the smaller the slump movement process under gravity. The error between the simulation analysis of the slump of the yield stress and the empirical formula is less than 10%. The effect of density on slump is converted between relative gravity and yield stress. Under the same yield stress, the smaller the density is, the smaller the gravity is. Therefore, the larger the density is, the larger the expansion and slump are. The influence of power law index on slump simulation is to affect the initial motion state. At this time, the acceleration is the largest, the shear rate is the largest, and the corresponding shear stress changes the most. With the extension of the simulation time, the movement is gradually gentle, and the shear stress fluctuates up and down at the yield stress. The influence of the final slump and the expansion degree is close to each other. The slump simulation analysis process of spherical coarse aggregate and non-spherical coarse aggregate is compared. The results show that the slump and expansion of spherical coarse aggregate are larger than those of non-spherical particles.
4. Concrete pumping model is established and the pumping pressure test is carried out. The simulation analysis results are compared with the experimental results. The consistency of the simulation initial value and the trend of the intermediate process is obtained. Considering that the rheological parameters of the boundary layer will change during the pumping process, the boundary layer characteristics can be added in the follow-up study to more accurately simulate the rheological properties of the concrete during the pumping process.

## Data Availability

The data used to support the findings of this study are available from the corresponding author upon request.
